# Cluster headache and Bipolar disorders: Phenotypical Overlap and Comorbidity.

**DOI:** 10.1192/j.eurpsy.2025.1212

**Published:** 2025-08-26

**Authors:** M. A. Riedinger, R. B. Brandt, S. E. Knapen, E. J. Giltay, N. J. van der Wee, R. Fronczek, M. de Leeuw

**Affiliations:** 1 Psychiatry, Leiden University Medical Center; 2 Outpatient clinic for mental disability and psychiatry, GGZ Rivierduinen; 3 Neurology, Leiden University Medical Center, Leiden; 4 Public Health and Primary Care, Leiden University Medical Center, The Hague; 5 Sleep-Wake Center, Stichting Epilepsie Instellingen Nederland (SEIN), Heemstede; 6 Outpatient clinic for Bipolar Disorder, GGZ Rivierduinen, Leiden, Netherlands

## Abstract

**Introduction:**

Cluster headache (CH) and bipolar disorder (BD) are cyclic disorders with shared clinical aspects, e.g. disturbed sleep patterns, response to lithium and a tendency to use illicit drugs.

**Objectives:**

We investigated comorbidity of BD in patients with CH, prevalence of BD amongst CH family members, circadian aspects and use of illicit drugs.

**Methods:**

Patients from the Leiden University Cluster headache neuro-Analysis program (LUCA) were screened cross-sectionally for BD online, using the Mood Disturbance Questionnaire (MDQ-NL) and Altman Self Rating Scale (ASRM-NL) and, if indicated, further evaluated with the Mini international neuropsychiatric interview (MINI). Circadian aspects were analyzed using the Circadian Type Inventory (CTI) and the Munich Chronotype Questionnaire (MCTQ).

**Results:**

The life-time prevalence of BD in patients suffering from CH was 6.5%. Patients with comorbid BD (CH+BD) were more likely to have family members with BD (standardized OR= 1.50, 95% CI=1.15;1.95, p=0.003) and were more likely to suffer from chronic CH (OR=3.05 95% CI=1.18;7.87, p=0.02) after adjustment for age and sex. They also more often used illicit drugs, compared to patients with only CH. Circadian type and chronotype did not differ between the two groups after adjustment for confounders.

**Conclusions:**

There is a high prevalence of BD in CH patients. CH+BD patients are more likely to have a positive family history for BD. CH+BD patients are more likely to suffer from chronic CH and more often use illicit drugs. In clinical practice it is important to screen for BD when treating patients with CH.

**Disclosure of Interest:**

None Declared

